# Supplementary figures and images for: Healthcare professionals’ perceptions and recommendations regarding adolescent vaccinations in Georgia and Tennessee during the COVID-19 pandemic: A qualitative research
Source: PLoS One. 2022 Nov 18;17(11):e0277748. doi: 10.1371/journal.pone.0277748 (PMC9674128; doi:10.1371/journal.pone.0277748)

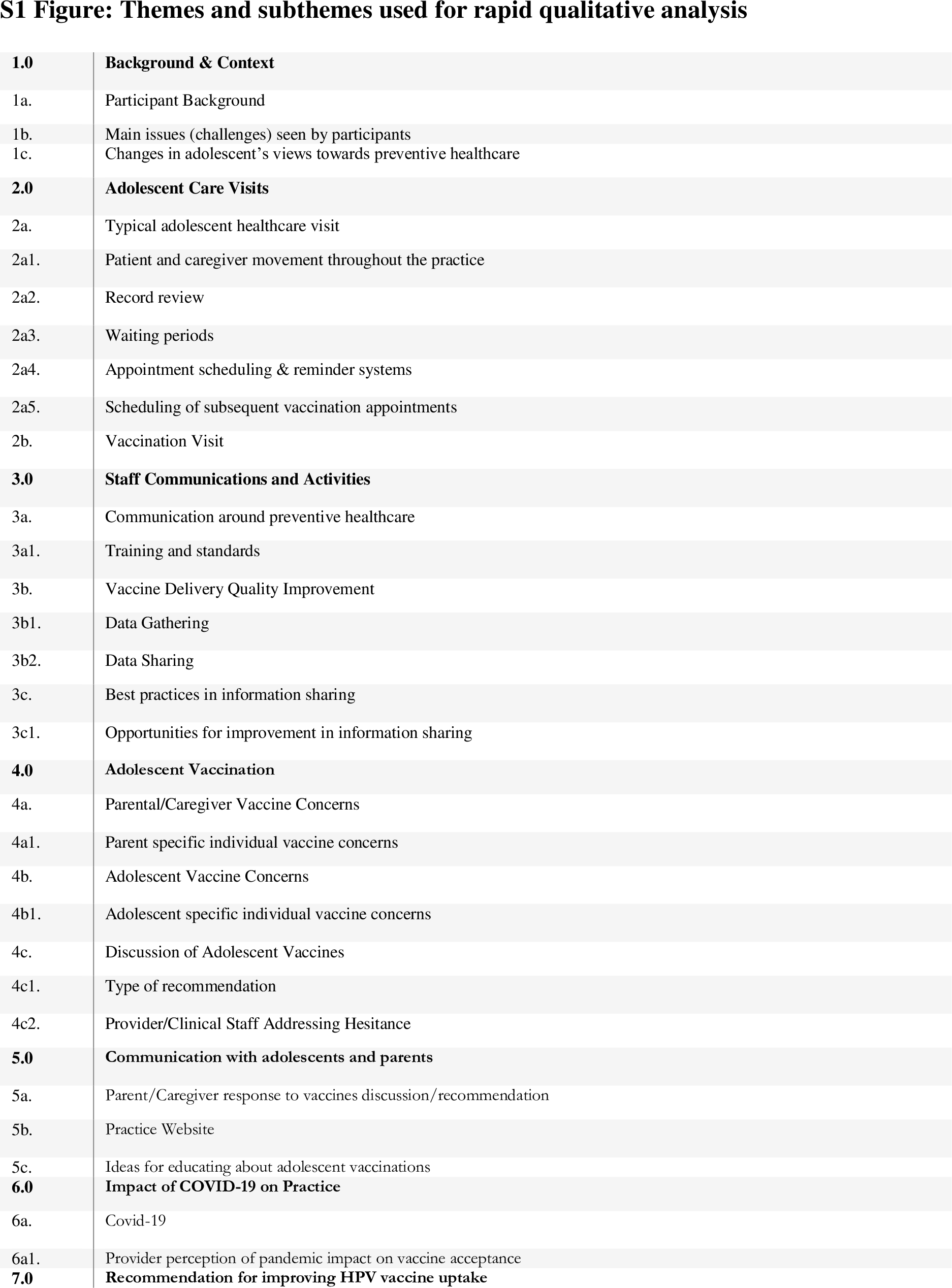

Supplement: S1 Fig — (TIF) [file pone.0277748.s001.tif]
